# Development of a Xeno-Free Autologous Culture System for Endothelial Progenitor Cells Derived from Human Umbilical Cord Blood

**DOI:** 10.1371/journal.pone.0075224

**Published:** 2013-09-24

**Authors:** Sung-Hwan Moon, Sun-Mi Kim, Soon-Jung Park, Hojin Kim, Daekyeong Bae, Yong-Soo Choi, Hyung-Min Chung

**Affiliations:** 1 Department of Stem Cell Biology, Konkuk University School of Medicine, Seoul, South Korea; 2 Department of Applied Bioscience, CHA University, Seongnam-si, South Korea; 3 CHA Bio & Diostech Co., Ltd. Seoul, South Korea; University of Kansas Medical Center, United States of America

## Abstract

Despite promising preclinical outcomes in animal models, a number of challenges remain for human clinical use. In particular, expanding a large number of endothelial progenitor cells (EPCs) in vitro in the absence of animal-derived products is the most critical hurdle remaining to be overcome to ensure the safety and efficiency of human therapy. To develop in vitro culture conditions for EPCs derived from human cord blood (hCB-EPCs), we isolated extracts (UCE) and collagen (UC-collagen) from umbilical cord tissue to replace their animal-derived counterparts. UC-collagen and UCE efficiently supported the attachment and proliferation of hCB-EPCs in a manner comparable to that of animal-derived collagen in the conventional culture system. Our developed autologous culture system maintained the typical characteristics of hCB-EPCs, as represented by the expression of EPC-associated surface markers. In addition, the therapeutic potential of hCB-EPCs was confirmed when the transplantation of hCB-EPCs cultured in this autologous culture system promoted limb salvage in a mouse model of hindlimb ischemia and was shown to contribute to attenuating muscle degeneration and fibrosis. We suggest that the umbilical cord represents a source for autologous biomaterials for the in vitro culture of hCB-EPCs. The main characteristics and therapeutic potential of hCB-EPCs were not compromised in developed autologous culture system. The absence of animal-derived products in our newly developed in vitro culture removes concerns associated with secondary contamination. Thus, we hope that this culture system accelerates the realization of therapeutic applications of autologous hCB-EPCs for human vascular diseases.

## Introduction

Coronary artery ischemia, diabetic ulcers, and myocardial infarction are common ischemic diseases threatening human lives that develop due to the dysfunction of endothelial progenitor cells (EPCs) [1], [2]. The transplantation of healthy EPCs to compensate for their dysfunctional counterparts has been reported to yield promising improvements in various animal models of ischemic disease [3], [4], [5]. Thus, EPCs are now widely considered to be an effective therapeutic agent for treating vascular diseases.

In clinical settings, to optimize therapeutic efficacy and to ensure safety, EPCs must be 1) derived from an autologous source to prevent immune rejection, 2) non-tumorigenic, 3) free of animal-derived products, and 4) secured in sufficient numbers. Autologous EPCs derived from sources such as peripheral blood, bone marrow and adipose tissue fulfill most of these criteria, but challenges remain for in vitro expansion to clinical levels [6], [7]. Autologous EPCs have also been reported to be isolated from cord blood (CB), which is discarded at birth [8], [9]. CB-EPCs exhibit the main characteristics of EPCs in parallel to those derived from adult sources and have also been reported to be therapeutically viable in repairing damaged vascular tissues [10], [11], [12].

The main advantage of CB-EPCs over adult tissue-derived EPCs is their superior proliferative capacity, indicating that the number required for human clinical application (10^9^ cells) could be achieved in vitro [13], [14]. To expand EPCs to the required numbers, routine laboratory practice involves the use of animal-derived extracellular matrices (ECMs) and serum, such as fetal bovine serum (FBS) [15], [16], [17]. Such products are easily obtainable and efficiently support the proliferative capacity of EPCs, but there exists a major pitfall in that the transmission of animal-derived pathogens could prove lethal for human clinical applications [18]. Human-derived ECMs and serum are available as replacements, but these products are unlikely to be 1) autologous, 2) available in large quantities, and 3) consistent between batches [19], [20].

In this study, we developed a xeno-free autologous culture system for the in vitro expansion of CB-EPCs. From an umbilical cord (UC), we removed cord blood from which EPCs (CB-EPCs) were isolated that exhibited the typical characteristics of EPCs. In addition, extracts (UCE) and collagen (UC-collagen) were isolated from the same UC and used as the serum and ECM, respectively, for the in vitro expansion of the EPCs. Both UCE and UC-collagen supported proliferation and the main characteristics of CB-EPCs in a manner comparable to that of commercialized collagen and FBS. In addition, the therapeutic potential of CB-EPCs was confirmed after transplantation in a mouse model of hindlimb ischemia. Through our data, we have demonstrated that CB-EPCs cultured in our developed culture system fulfill all of the aforementioned criteria for successful clinical outcomes of EPC transplantation.

## Materials and Methods

### Ethics Statement

Human UC and UC blood samples were donated by healthy volunteers at CHA General Hospital (Seoul, South Korea) and used within 24 hours. This study was approved by the Institutional Review Board of CHA Stem Cell Institute in CHA University. All information pertaining to subjects and all human samples were used in compliance with Korean legislation, and all human participants gave written informed consent [21].

### Purification of collagen

To purify collagen from human UCs, we dissociated the UC tissues into 1-cm pieces using sterile surgical scissors and washed the pieces in distilled water. For virus inactivation, the dissociated tissues were soaked in 70% EtOH at 4°C for 24 hours. The tissue was then washed in distilled water and stirred in 3% H_2_O_2_ on a magnetic stirrer at 4°C for 24 hours. To crush the tissues, we homogenized them in 0.5 M acetic acid and transferred them into pepsin (Sigma-Aldrich, St. Louis, MO) at 4°C for 24 hours. The tissue suspensions were centrifuged at 10,000 rpm for 30 minutes at 4°C after being brought to pH 7 with NaOH for pepsin inactivation and desalting. The supernatant proteins were then precipitated with NaCl for 12 hours. The mixture was clarified at 10,000 rpm for 30 minutes at 4°C, and the supernatant was subsequently desalted and concentrated using an ultrafiltration system [22]. Finally, the collagen was lyophilized, and the purified collagen was analyzed using the hydroxyproline method [23].

### Extractions of human umbilical cord extract (UCE)

Human UC tissues were dissociated into small pieces (approximately 1 cm in length) using sterile surgical scissors and stirred in PBS (HyClone, Logan, UT) on a magnetic stirrer for 24 hours at 4°C. The stirred suspensions were centrifuged at 4,500 rpm for 15 minutes at 4°C, and the supernatants were collected and labeled “umbilical cord extract (UCE)”. Bradford reagent (Bio-Rad Laboratories, Hercules, CA) was used for protein assays. Fresh UCE was frozen at −20°C after lyophilization for future use.

### SDS-PAGE analysis for purity of UC-collagen and UCE

To analyze the purity of UC-collagen and UCE, we performed sodium dodecyl sulfate-polyacrylamide gel electrophoresis (SDS-PAGE) analysis using the Laemmli method [24] with 6% native-polyacrylamide gels and 5% stacking gels at room temperature. After heating at 95°C for 5 minutes, the samples (20 µg) were applied to sample wells and electrophoresed along with molecular weight markers in a gel electrophoresis system (Bio-Rad). The gel was stained for protein with 0.1% Coomassie Brilliant Blue R-250 and de-stained in 10% methanol and 10% acetic acid. Using SDS-PAGE analysis, we confirmed that the purification of UC-collagen type I compared favorably with the two commercially available collagen type I products (StemCell Technologies, Vancouver, Canada and Bioland, Chonan, South Korea) through the appearance of triple-helical proteins during electrophoretic mobility (Figure S1A). In addition, the identification of UCE was compared with fetal bovine serum (FBS; HyClone) (Figure S1B).

### Derivation of endothelial progenitor cells derived from human umbilical cord blood (hCB-EPCs)

The derivation and culture of hCB-EPCs were performed as described previously [9]. Briefly, mononuclear cells (MNCs) were first isolated by density gradient centrifugation using Histopaque-1083 (Sigma-Aldrich) from fresh UCB. MNCs were plated on collagen-coated tissue culture flasks at a density of 4–6×10^6^/mL in EBM2 medium (Clonetics, Walkersville, MD) with 5% FBS and a cytokine cocktail (SingleQuot, Clonetics) that included vascular endothelial growth factor, fibroblast growth factor, insulin-like growth factor, hydrocortisone, ascorbic acid, and heparin, as previously described [25]. Nonadherent cells were removed after 4 days of culture, and the medium was replaced. The cells were maintained for 7 days and used as an enriched EPC population after characterization.

### Analysis of cell attachment and proliferation rate

To assay cell attachment and proliferation, a total of 3×10^5^ hCB-EPCs were seeded on non-coated or collagen-coated plates and cultured in DMEM or EGM2 medium supplemented with either UCE or FBS (HyClone). After harvesting, the cells were counted using a hemocytometer after trypan blue (Sigma-Aldrich) staining. Attachment and proliferation rates were calculated as a percentage of trypan blue-stained viable cells from the harvested cells.

### Immunohistochemical analysis

The cells were fixed with 4% paraformaldehyde and permeabilized with 0.1% Triton X-100 in PBS (Gibco, Gaithersburg, MD) for 5 minutes. After treatment with 1% normal goat serum for 30 minutes, the cells were incubated with PECAM (Millipore, Billerica, MA, USA) and vWF (Abcam Inc., Cambridge, MA, USA) antibodies for 12 hours at 4°C. The cells were washed with PBS and then incubated with rhodamine- and FITC-conjugated secondary antibodies (Molecular Probes Inc., Eugene, OR, USA) for 1 hour. Cells were then washed with PBS and stained with DAPI (Invitrogen, Grand Island, NY, USA). All images were analyzed using an LSM 510 META confocal microscope (Carl Zeiss Inc., Oberkochen, Germany).

### Laser Doppler imaging analysis

The laser Doppler imaging analysis was performed as previously described [4]. A laser Doppler perfusion imager (Moor Instruments, Devon, UK) was used to measure blood flow in the hind limbs on days 0, 7, 14, and 28 post-treatment. Digital color-coded images were analyzed to quantify the blood flow in the region from the knee joint to the toe, and the mean perfusion values were calculated.

### Histological and immunohistochemical analysis

For tissue staining, we euthanized the mice and retrieved ischemic limb tissues at 4 weeks post-transplantation. Specimens were fixed in 4% paraformaldehyde (PFA) with a graded ethanol series and embedded in paraffin. The specimens were sliced into 4-µm sections and stained with hematoxylin and eosin. Masson's trichrome collagen staining was performed to examine fibrosis in the ischemic tissues. Normal limb muscle that had not undergone surgery was used as a positive control. For immunofluorescent staining, 1 day before euthanasia, we injected RITC-conjugated lectin (Sigma-Aldrich) through the tail vein. The specimens were embedded in O.C.T. Compound (Sakura Finetek USA Inc.), frozen, and cut into 10- to 20-µm-thick sections at −20°C. The tissue sections were immunofluorescently stained with anti-human nuclear antigen (HNA, Chemicon) and visualized with FITC-conjugated anti-mouse IgG (Molecular Probes Inc). All fluorescence images were acquired using an LSM 510 META confocal microscope (Carl Zeiss Inc.).

### FACS analysis

Flow cytometric staining and analyses were performed as previously described [4]. Briefly, hCB-EPCs were resuspended in 100 µL rinsing buffer and incubated with phycoerythrin (PE)-conjugated mouse anti-human CD31, CD34, CD105, CD90, and CD45 (BD Biosciences) antibodies. After washing, the cells were analyzed with a FACSCalibur flow cytometer equipped with the Cell Quest software (BD Biosciences). Flow cytometric data were analyzed using appropriate controls with proper isotype-matched IgG and unstained controls.

### Mouse limb ischemia and cell transplantation

All animal care and experimental procedures were performed under the approval of the animal care committees of CHA University (IACUC No. 08_003). Male athymic nude mice (6 to 7 weeks old, body weight 25 to 30 g; Charles River Laboratories International, Inc., Wilmington, MA, USA) were anesthetized with ketamine (100 mg/kg) and Rompun (25 mg/kg). The femoral artery and its branches were ligated through a skin incision with 4–0 silk (Ethicon, Somerville, NJ, USA). The external iliac artery and all of the anterior arteries were then ligated. The femoral artery was excised from its proximal origin as a branch of the external iliac artery to the distal point where it bifurcates into the saphenous and popliteal arteries [26]. To determine the therapeutic efficacy of our cells in hindlimb ischemia, hCB-EPCs (3.0×10^6^ cells per mouse (n = 6)) from the newly developed culture system were suspended in 100 µl of PBS and injected intramuscularly into four sites of the gracilis muscle in ischemic limbs. The control group (n = 6) was injected with PBS only.

### In vitro tubular structure assay

For in vitro functional analysis of hCB-EPCs, 5×10^4^ cells were plated on Matrigel-coated (BD Biosciences, Bedford, MA) well plates and subsequently incubated at 37°C in 5% CO_2_ for 12 hours. The formation of tubular structures was measured and analyzed using a Ti fluorescence microscope (Nikon, Chiyoda-ku, Japan) [27].

### Statistical analyses

Quantitative data are expressed as the mean ± standard deviation. Statistical analyses were performed with one-way analysis of variance (ANOVA) using the Statistical Package for the Social Sciences (SPSS) software (SPSS Inc., Chicago, IL). A value of p<0.05 was considered to be significant.

## Results

### Schematics for the development of a xeno-free autologous culture system for hCB-EPCs

**Figure 1:**
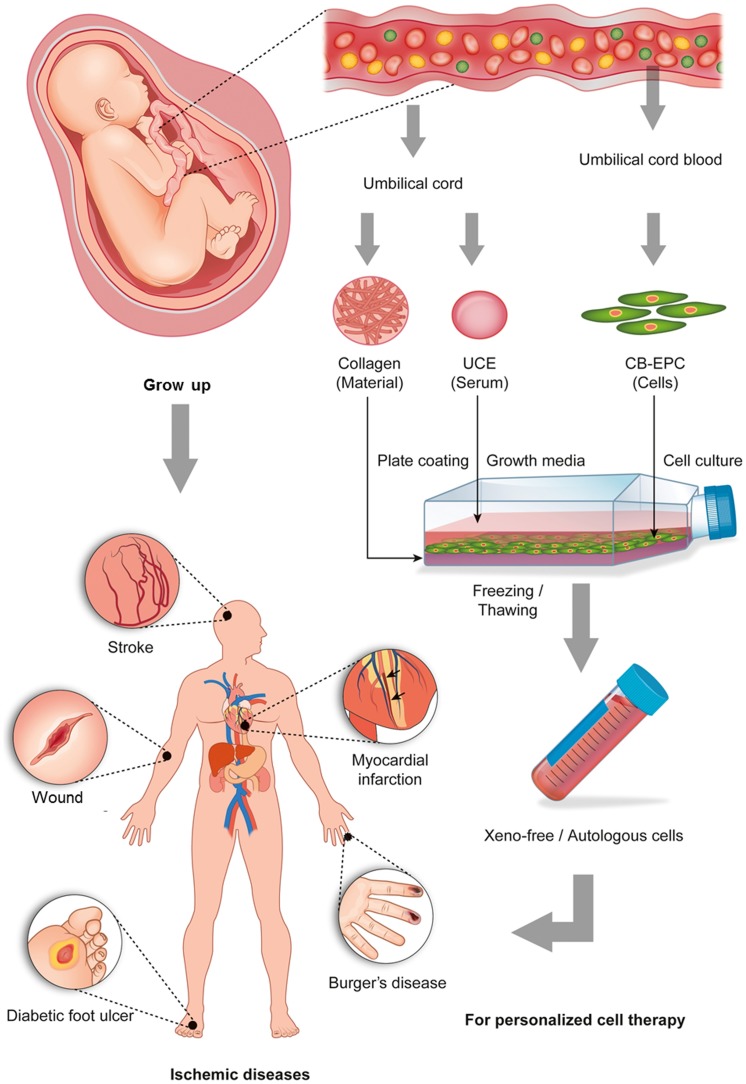
Overall strategy of the autologous culture system using human UC tissue and cord blood.

EPCs are isolated from the UC blood of a newborn baby and cultured in vitro with the autologous ECM (collagen) and serum (umbilical cord extract) isolated from the UC of the same newborn baby. EPCs expand in this xeno-free autologous culture system, which can be cryopreserved for future use by the baby (Figure 1).

### Effects of UC-collagen on the attachment and proliferation of hCB-EPCs

**Figure 2:**
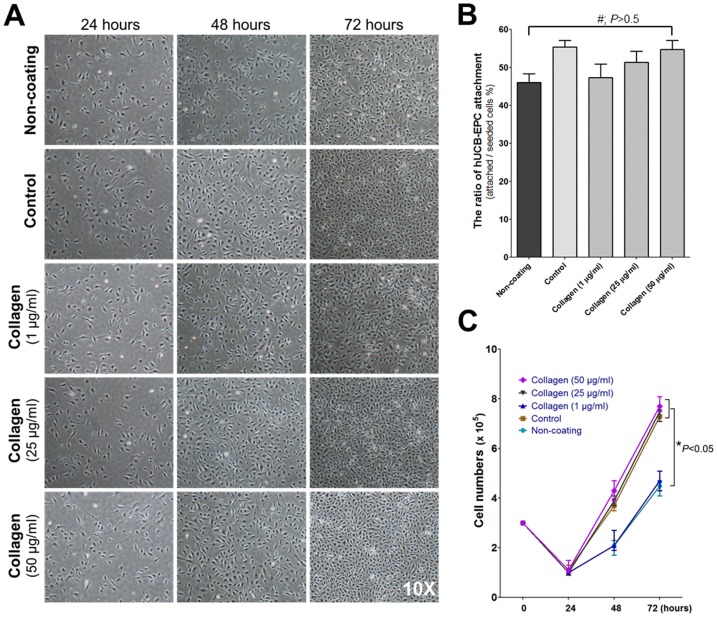
The attachment and proliferation of hCB-EPCs on UC-collagen. (A) Attached hCB-EPCs in the non-coated group, control group and UC-collagen (1, 25, and 50 µg/ml) groups imaged using optical microscopy (10×). (B) Quantification of attached hCB-EPCs. (C) The proliferation of hCB-EPCs on various collagen-coated plates (*p<0.05).

A total 3×10^5^ hCB-EPCs were seeded on non-coated plates, plates coated with DW containing 0.02% (v/v) commercialized collagen as a positive control (StemCell Technologies), and plates coated with varying concentrations of UC-collagen (1, 25, and 50 µg/ml). The cells in all groups were cultured in DMEM supplemented with 10% FBS for 3 days to examine cell attachment and proliferation. Through microscopic observation, it appeared that the cells in all groups successfully attached 24 hours post-seeding (Figure 2A, left panel), and their ability to attach to the surface of the plates in the first 24 hours was not significantly different between the groups tested (Figure 2B). Regarding the proliferative capacity of hCB-EPCs on the different coating materials, we observed an initial dip in proliferation in the first 24 hours post-seeding in all groups (Figure 2C). However, hCB-EPCs cultured on UC-collagen (25 and 50 µg/ml) and collagen proliferated at a constant rate, reaching approximately 8×10^5^ cells 72 hours post-seeding (Figure 2C). hCB-EPCs cultured on the non- and UC-collagen (1 µg/ml)-coated plates also proliferated, but at a much slower rate, yielding approximately 4×10^5^ cells 72 hours after plating (Figure 2C). This apparent difference in cell proliferation was also evident through microscopic observation, as hCB-EPCs cultured on UC-collagen (25 and 50 µg/ml) and collagen became confluent 72 hours post-seeding, whereas those cultured on non- and UC-collagen (1 µg/ml)-coated plates appeared to be less confluent (Figure 2A, right panel). These data suggest that appropriate concentrations of UC-collagen (25 and 50 µg/ml in this instance) sufficiently support the proliferation of hCB-EPCs in a manner similar to that obtained with commercially available collagen.

### UCE efficiently supports the proliferation of hCB-EPCs

**Figure 3:**
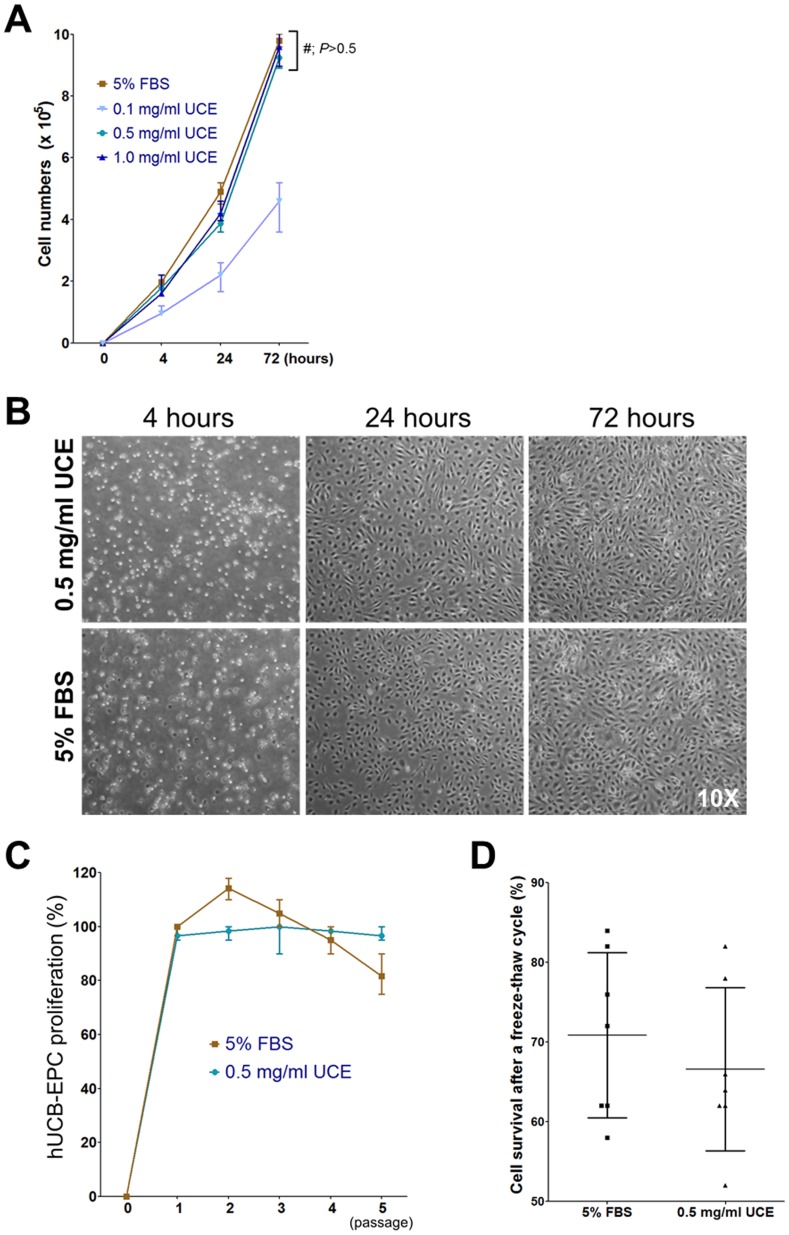
Effects of UC-collagen and UCE for hCB-EPC culture. (A) Proliferation capacity of hCB-EPCs on UC-collagen and cultured in 5% FBS or varied concentrations of UCE (0.1, 0.5, and 1.0 mg/ml) (*#<0.05). (B) Attachment and proliferation capacity of 0.5 mg/ml UCE medium compared with 5% FBS medium on UC-collagen-coated plates as shown in optical microscopy images (10×). (C) Quantification of hCB-EPC proliferation at different passage numbers. (D) Measurement of hCB-EPC survival cultured in 0.5 mg/ml UCE- or 5% FBS-containing medium after a freeze-thaw cycle.

hCB-EPCs are routinely cultured in EGM2 medium (EBM2 medium (Clonetics) supplemented with a cytokine cocktail (SingleQuot; Clonetics)) and with 5% FBS because of the ability of this preparation to support hCB-EPC proliferation [25]. However, the use of animal byproducts is considered to be clinically unsuitable in the culture of cells intended for human use due to potential cross-species contamination. To examine whether UCE could replace animal-derived FBS, we performed a comparative study by culturing hCB-EPCs in EGM2 medium supplemented with 5% FBS or UCE (0.1, 0.5, and 1.0 mg/ml) for 3 days on 50 µg/ml UC-collagen-coated plates. Our quantitative analysis revealed no significant differences in cell proliferation when viable cells were counted at 4, 24, and 72 hours post-seeding in both 5% FBS and UCE (0.5 and 1.0 mg/ml) culture conditions. However, cells cultured in medium supplemented with 0.1 mg/ml UCE demonstrated significantly impaired proliferation (Figure 3A). Microscopic observations also revealed no visual differences in terms of the cells' ability to attach and proliferate in both 5% FBS and 0.5 mg/ml UCE culture conditions (Figure 3B). Next, we compared the proliferation of hCB-EPCs in EGM2 supplemented with 5% FBS or 0.5 mg/ml UCE. The proliferation of hCB-EPCs at each passage was normalized to that of first-passage hCB-EPCs cultured with 5% FBS supplementation. The proliferation of hCB-EPCs cultured in the presence of FBS demonstrated passage-to-passage variation, as the cells demonstrated reduced proliferation after the second passage. In contrast, the proliferation of hCB-EPCs cultured with UCE was shown to be more consistent between passages (Figure 3C). The survival rate of hCB-EPCs cultured in 0.5 mg/ml UCE after a freeze-thaw cycle was measured and was not significantly different from the survival rate of hCB-EPCs cultured with FBS (Figure 3D). These observations show that UCE can replace animal-derived FBS for in vitro culture without reducing cell proliferative capacity.

### In vitro characterization of hCB-EPCs cultured in UC-collagen and UCE

**Figure 4:**
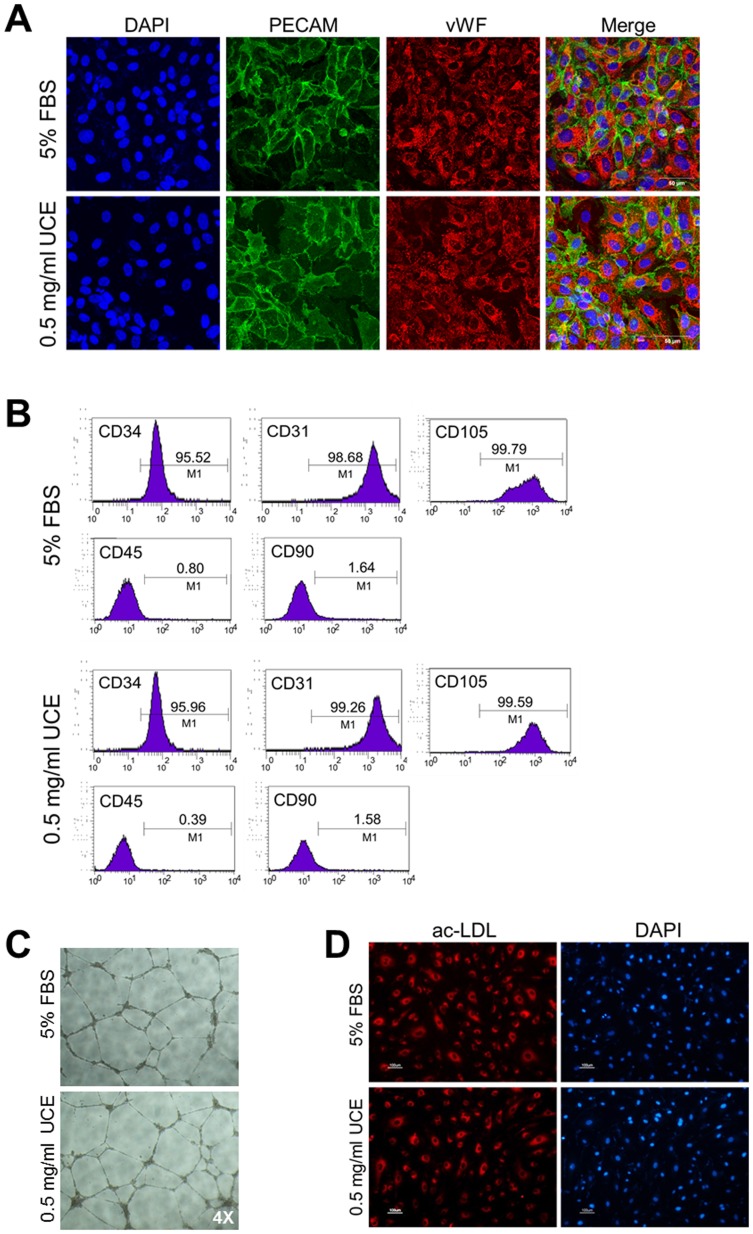
Phenotypic characterization and functional analysis of hCB-EPCs. (A) Cultured hCB-EPCs grown in 5% FBS or 0.5 mg/ml UCE medium showing endothelial-specific marker staining for PECAM and vWF. (B) FACS analysis of cultured hCB-EPCs for the expression of the endothelial markers CD34, CD31, and CD105; the mesenchymal marker CD90; and the hematopoietic maker CD45. (C) Capillary-like structures formed by hCB-EPCs in 5% FBS or 0.5 mg/ml UCE on Matrigel. (D) Acetylated LDL uptake and DAPI staining.

To determine whether culturing hCB-EPCs in UC-collagen and UCE altered their main characteristics and functional properties, we first performed immunocytochemistry using antibodies against the endothelial-specific markers PECAM and vWF. It was revealed that hCB-EPCs cultured in our novel culture system expressed high levels of PECAM and vWF compared with hCB-EPCs cultured in the standard culture condition (EGM2 medium supplemented with 5% FBS) (Figure 4A). In addition, FACS analysis revealed that the percentages of CD34-, CD31-, and CD105-positive cells were virtually the same between hCB-EPCs cultured under both culture conditions. We observed a low percentage of cells expressing CD45 and CD90 in our novel culture system, similar to the standard culture condition (Figure 4B). To examine the in vitro functional properties of hCB-EPCs cultured in our culture system, we plated them and hCB-EPCs cultured in the standard culture condition on Matrigel. Cells cultured in both culture systems formed vessel-like tubular structures (Figure 4C). Additionally, we confirmed the ability of hCB-EPCs to take up acetylated low-density lipoprotein (Ac-LDL) by monitoring Ac-LDL fluorescence (Figure 4D). Fluorescence was observed in almost all hCB-EPCs cultured under both conditions (Figure 4D). Taken together, our observations indicate that our novel culture system consisting of autologous ECM and serum does not alter the characteristics and functions of hCB-EPCs.

### hCB-EPCs improve blood perfusion and attenuate muscle degeneration in a mouse model of hindlimb ischemia

**Figure 5:**
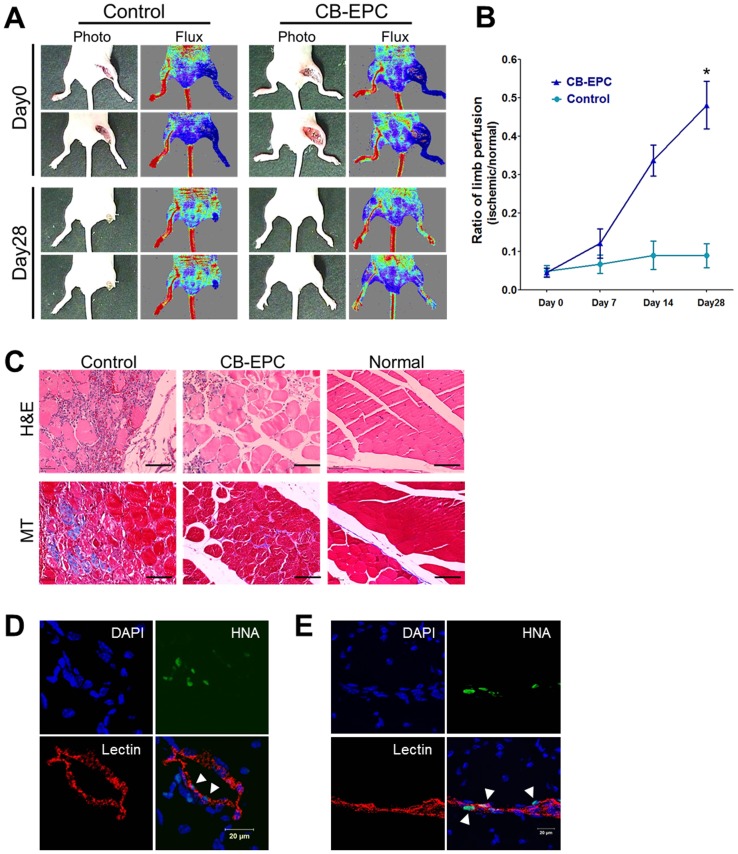
Improvement of blood flow in ischemic hindlimb post-transplantation of hCB-EPCs and histological analysis. (A) Representative laser Doppler imaging. Blood flow in an ultimately lost limb from the control group and a salvaged limb from the hCB-EPC-treated group. (B) Ratio of blood flow perfusion in the ischemic limb for each group (*p<0.05). (C) Histologic images of hematoxylin and eosin staining for the analysis of muscle degeneration; Masson's trichrome staining showed fibrosis in the ischemic region. (D) Engraftment of transplanted hCB-EPCs in ischemic regions.

To examine the therapeutic potential of hCB-EPCs cultured in our novel culture system, we first surgically generated a mouse model of hindlimb ischemia, which we verified by measuring blood perfusion at day 0 using a laser Doppler perfusion imaging system (Figure 5A). The control group, which received PBS, exhibited limb loss or foot necrosis 28 days after the injection, whereas the treated group demonstrated limb salvage (Figure 5A). The blood flow in the ischemic region remained low when measured at 7-day intervals for 4 weeks post-transplantation in the mice that received PBS. In contrast, we observed a non-significant increase in blood perfusion 7 days after transplanting hCB-EPCs compared with the PBS control group (Figure 5B). Blood perfusion continued to increase, and when measured again on day 28 post-transplantation, it was approximately 5 times higher than the perfusion observed in the mice that had received PBS (Figure 5B, *P<0.05). Histological examinations of ischemic limbs harvested 28 days after the injection treatment revealed that hCB-EPC transplantation protected limb muscles against necrotic damage caused by ischemia. Hematoxylin and eosin staining of the control group (PBS injection) showed massive muscle degeneration in the ischemic regions (Figure 5C). In contrast, muscles in the ischemic limbs of mice in the hCB-EPC transplantation group were protected after cell transplantation (Figure 5C). In addition, Masson's trichrome staining revealed markedly reduced fibrosis in the hCB-EPC transplantation groups compared with the PBS-injected control group (Figure 5C). Next, we performed an immunohistochemical analysis to detect the cell engraftment of injected hCB-EPCs in ischemic regions. One day before euthanasia, the mice were injected with RITC-conjugated lectin to label all vessels in the ischemic regions. Certain lectin-positive vessels in the ischemic regions of mice in the hCB-EPC transplantation group were also positive for human nuclear antigen (HNA) (Figure 5D, white arrowhead). Furthermore, HNA-positive transplanted hCB-EPCs were found in capillary-like structures near muscle tissue in the ischemic region and incorporated into vessels between muscle tissues (Figure 5E, white arrowhead). In contrast, lectin-positive vessels in the ischemic region of the PBS-injected group were negative for HNA (data not shown). These data suggest that the transplanted hCB-EPCs not only survived for 4 weeks by incorporating into the host vascular structure but also induced neovascularization in the ischemic region in concert with host cells.

## Discussion

In this study, we have developed a xeno-free autologous culture system for CB-EPCs for potential human clinical use. The animal-derived products that typically support the proliferation of CB-EPCs are replaced with UC-collagen and UCE isolated from the same UC for the expansion of cell numbers in vitro. We believe that the culture system developed in this study paves the way for the development of an efficient and safe treatment for human vascular disease. The fact that autologous collagen and serum can be obtained from the patient circumvents many problems associated with cell replacement therapy. In addition, UC-collagen and UCE can be cryopreserved along with CB-EPCs; therefore, when the time comes for these cells to be used for clinical purposes, they can then be cultured in this xeno-free autologous culture system.

The therapeutic value of EPCs for the improvement of vascular diseases has been widely recognized for decades. Translational studies have shown that EPCs promote angiogenesis and neovascularization, improving blood flow in ischemic injury sites [10]. The fact that autologous EPCs are readily available from adult tissues suggests that the development of an autologous cell therapy is imminent [28]. Autologous cell therapy is advantageous in that the cells are less prone to immune rejection, in contrast to those derived from other sources, such as human pluripotent stem cells (hPSCs) [29], [30], [31]. Despite this advantage, the actual number of autologous EPCs extractable from adult tissues is very low; thus, EPCs are often subject to in vitro expansion to reach the large numbers required for cell therapy [28]. EPCs at this stage are very susceptible to contamination because they are routinely cultured in the presence of animal-derived products, such as ECM and serum [32]. Although human-originated products may serve as replacements, the introduction of outside sources, including allogenic materials, can diminish the immunogenic advantages that the autologous EPCs possess [33]. In this study, we report that extracts and collagen isolated from UC tissue efficiently support the proliferation of CB-EPCs derived from the same UC. This innovative culture system overcomes the problems associated with immunogenicity and contamination from animal-derived products, making it viable for human clinical use.

The data presented in this study demonstrate that UC-collagen and UCE are plausible autologous biomaterials to replace conventionally used commercialized collagen and FBS for the expansion of CB-EPCs to the numbers required for transplantation. UC-collagen and UCE support CB-EPC proliferation as efficiently as commercialized collagen and FBS (Figure 3). To our surprise, CB-EPCs cultured with UC-collagen and UCE exhibited a stable proliferative capacity passage after passage, whereas the proliferation of those cells cultured in the conventional condition decreased after the first passage (Figure 3C). This observation suggests that expanding CB-EPCs to a clinical level can be achieved more efficiently with autologous biomaterials, which could potentially reduce the cost of the overall treatment. The fact that the characteristics and functionality of CB-EPCs proliferated in UC-collagen and UCE are unaltered increases the chance of positive clinical outcomes after in vivo transplantation. Indeed, after the injection of CB-EPCs, mouse models of hindlimb ischemia demonstrated limb salvage in the ischemic regions, substantially reduced fibrosis, and attenuated muscle degeneration (Figure 5C). In conclusion, UCE and UC-collagen are extracted from the umbilical cord of a newborn baby. These were used to establish a xeno-free culture system for EPC derived from the cord blood of the same umbilical cord. CB-EPC cultured in the xeno-free system demonstrated therapeutic potential for vascular diseases in animal models. We report an establishment of the xeno-free culture system for an autologous transplantation of patient-derive CB-EPC.

## Supporting Information

Figure S1 **SDS-PAGE analysis of the purity of UC-collagen type I and UCE.** (A) Type 1 human placental collagen (StemCell Technologies, lane 1), Type 1 Atelocollagen from rat tail tendon (Bioland Ltd., lane 2), and type 1 collagen from human UC tissue (lane 3). M; marker. (B) FBS (HyClone, lane 1) and UCE (lane 2).
(DOC)
