# Supplementary figures and images for: Development of a Xeno-Free Autologous Culture System for Endothelial Progenitor Cells Derived from Human Umbilical Cord Blood
Source: PLoS One. 2013 Sep 24;8(9):e75224. doi: 10.1371/journal.pone.0075224 (PMC3782462; doi:10.1371/journal.pone.0075224)

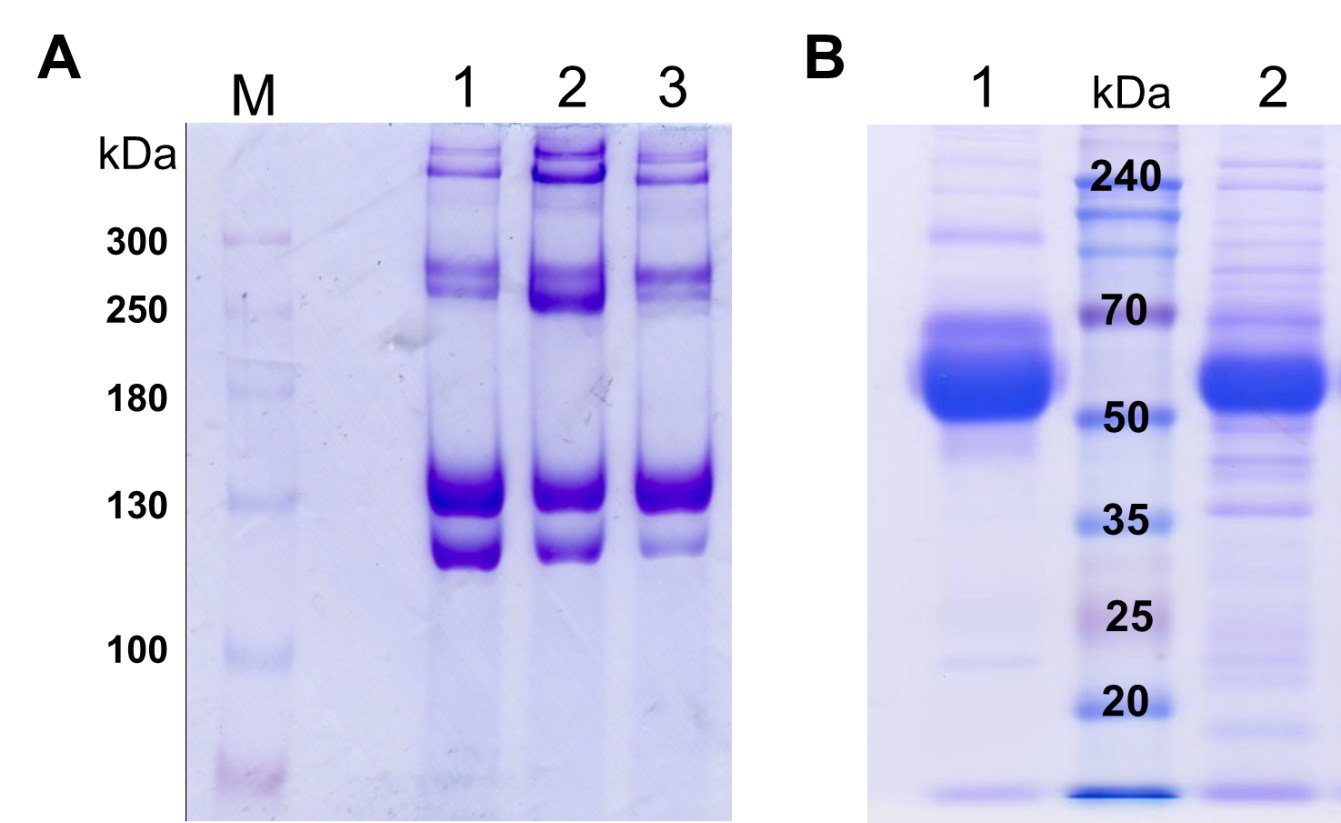

Supplement: Figure S1 — SDS-PAGE analysis of the purity of UC-collagen type I and UCE. (A) Type 1 human placental collagen (StemCell Technologies, lane 1), Type 1 Atelocollagen from rat tail tendon (Bioland Ltd., lane 2), and type 1 collagen from human UC tissue (lane 3). M; marker. (B) FBS (HyClone, lane 1) and UCE (lane 2). (DOC) [file pone.0075224.s001.doc]
